# Supplementary material for: Mental Health Literacy Intervention on Help-Seeking in Athletes: A Systematic Review
Source: Int J Environ Res Public Health. 2020 Oct 4;17(19):7263. doi: 10.3390/ijerph17197263 (PMC7579198; doi:10.3390/ijerph17197263)
Supplement: Supplementary file 1 [file ijerph-17-07263-s001.zip › ijerph-906977-suppl/Supplementary 2.doc]

Supplementary 2. Original search strategy for each database

1. Search strategy for the databases: PsycINFO and Scopus

Combined keyword searching were used: “mental health literacy OR mental health awareness OR mental health knowledge OR mental health first aid OR mental health service OR mental health strategy OR mental health promotion OR mental health education OR mental health prevention” AND “sport organisation OR sport club OR sport school OR sport setting OR sport training” AND “athlete OR player OR sportsman” AND “intervention OR training OR trial OR programme”.

2. Search strategy for the database: PubMed,

All fields with the following keywords were search: “mental health literacy OR mental health awareness OR mental health knowledge OR mental health first aid OR mental health service OR mental health strategy OR mental health promotion OR mental health education OR mental health prevention” AND “sport organisation OR sport club OR sport school OR sport setting OR sport training” AND “athlete OR player OR sportsman“ AND “intervention OR training OR trial OR programme”.

3. Search strategy for the database: Web of science

TS = (mental health literacy OR mental health awareness OR mental health knowledge OR mental health first aid OR mental health service OR mental health strategy OR mental health promotion OR mental health education OR mental health prevention) AND (sport organisation OR sport club OR sport school OR sport setting OR sport training) AND (athlete OR player OR sportsman) AND (intervention OR training OR trial OR programme).

Language limits in English. All document types, all years (1980-2020), and all citation indexes in the Web of Science Core Collection were searched.

4. Search strategy for the database: SPORTDiscus.

Combined keyword searching were used: “mental health literacy OR mental health awareness OR mental health knowledge OR mental health first aid OR mental health service OR mental health strategy OR mental health promotion OR mental health education OR mental health prevention” AND “sport organisation OR sport club OR sport school OR sport setting OR sport training” AND “athlete OR player OR sportsman” AND “intervention OR training OR trial OR programme”.

Language limits in English. All publication, all publication type, all database subset, all country and all document type were searched.
